# Spraying performance and deposition characteristics of an improved air-assisted nozzle with induction charging

**DOI:** 10.3389/fpls.2024.1309088

**Published:** 2024-03-28

**Authors:** Huitao Zhou, Mingxiong Ou, Xiang Dong, Wang Zhou, Shiqun Dai, Weidong Jia

**Affiliations:** ^1^ School of Agricultural Engineering, Jiangsu University, Zhenjiang, China; ^2^ Key Laboratory of Plant Protection Engineering of Ministry of Agriculture and Rural Affairs, Jiangsu University, Zhenjiang, China

**Keywords:** electrostatic spraying, air-assisted electrostatic nozzle, charge-to-mass ratio, droplet deposition and penetration, multi-factor orthogonal experiment method

## Abstract

Electrostatic spraying technology can improve the efficiency of pesticide deposition on the surface of leaves and reduce the environmental pollution caused by pesticide drift, which has an important prospect in agricultural pesticide application. To improve the deposition and penetration of droplets in the crop canopy, we designed and optimized an air-assisted electrostatic nozzle and conducted the spraying performance experiment. Parameters, such as charge-to-mass ratio (CMR) and particle size, were tested and analyzed to obtain the suitable operating parameters of nozzle. The results proved that the improved air-assisted electrostatic nozzle has good atomization and chargeability. There is a good charging effect with a charging voltage of 3,000–5,000 V, the CMR increased 127.8% from 0.86 to 1.97 mC/kg as the charge voltage increases from 1,000 to 4,000 V, at an air pressure of 1.0 bar and liquid flow rate of 200 ml/min. Furthermore, we designed a multi-factor orthogonal experiment, which was conducted using a four-factor, three-level design to investigate the effects of operational parameters and canopy characteristics on droplet deposition and penetration. Analysis of variance (ANOVA) and F-test were performed on the experiment results. The results showed that the factor effect on droplet penetration, in descending order, was as follows: spray distance, leaf area index, air pressure, and air pressure × spray distance. The factor effect on abaxial leaf deposition, in descending order, was as follows: air pressure, spray distance, air pressure × charge voltage, spray distance × charge voltage, and charge voltage. For optimal droplet penetration and abaxial leaf deposition, option *A*_3_ *B*_1_ *D*_2_ (air pressure 1.5 bar, spray distance 0.2 m, charge voltage 2,500 V) is recommend. The spray nozzle atomization performance and deposition regulation were studied by experimental methods to determine the optimal values of operating parameters to provide a reference for electrostatic spray system development.

## 1 Introduction

Pesticide spraying is an important activity of crop production in modern agriculture (Appah et al., 2019a). It ensures the deposition of pesticide spray droplets on target surfaces to control pests and diseases, and contributes to productivity as well as the quality of the yield (Chambers et al., 2014; Ahmad et al., 2020). Conventional hydraulic spraying for pesticide application using coarse droplet and large flow rate has been recognized as inefficient and cannot adhere well to the surface of the target, which causes off-target losses of pesticide droplets and gives rise to high residues in crop products and soil (Zhou et al., 2021; Wang et al., 2022). The electrostatic spray is used for agricultural application as an innovative plant protection strategy to overcome the above shortfalls during the 20th century (Hoffmann et al., 2007; Esehaghbeygi et al., 2010; Martini et al., 2016). The droplets are charged by the electric field of the electrode, which are rapidly deposited on the plant surface by static electricity, airflow traction, and gravity. It has been confirmed that the electrostatic method of pesticide application can provide greater control of droplet transport, improve overall deposition (especially the abaxial surface of leaves) and uniform distribution due to “wrap-around” effect, hence, reduce the off-target drift of pesticide droplets and the quantities of applied chemical pesticides (Zhu et al., 1989; Patel, 2016; Zhou et al., 2018). However, electrostatic spray also has some problems in agricultural pesticide application. Short charge retention time, easy leakage of charge, and easy adsorption of charged droplets near the electrode (Patel et al., 2017; Zhou et al., 2018) may cause the electrostatic spray to be not effective.

The scientific and engineering contributions of numerous researchers throughout the 20th century have established both the fundamental basis and the technical implementation of reliable and spray-charging methods (Law, 2001). Bowen et al. elucidated that a charged droplet population generates electric fields in space to facilitate pesticide droplet deposition from both theoretical and experimental aspects, which enriched the basic theory of electrostatic spraying (Bowen et al., 1964). Law et al. used inductive charging for electrostatic spraying of pesticides and proposed that the combination of electrostatic and air-assisted spraying would contribute to the deposition and penetration of droplets (Law and Bowen, 1966; Law, 1983). Since the 21st century, further research on the application of electrostatic spraying in orchards, greenhouses, and agricultural aviation were carried out. Pascuzzi and Cerruto (2015) conducted a study on spray deposition in “tendone” vineyards using an ESS “150RB14” air-assisted electrostatic spray system. The results showed that electrostatic spraying effectively improved the density and uniformity of droplet deposition on the lower layer of the canopy, but reduced the penetration of droplets compared with non-electrostatic spraying. Gan-Mor et al. (2014) investigated the charging of a conventional hydraulic nozzle. The results showed that electrostatic charging with an air-assisted electrostatic spray system improved the deposition of droplets by 200% and 500% on the leaf undersides and the rear of grape clusters, respectively. These demonstrate the effectiveness of the electrostatic application method in improving droplet deposition on leaf surface (especially abaxial side) and droplet penetration in crop canopy with the air-assisted electrostatic spray system (Ferguson et al., 2016).

Many relevant studies on the performance and deposition characteristics of the electrostatic spray had been conducted by researchers. Most of these researches focus on the effects of charging parameters (such as charging method, electrode material and mounting position, charging voltage, and electrode polarity). Law and Thompson (1996) concluded that the most widely used method for charging agricultural sprays is induction charging. The closer the electrode is to the liquid sheet, the better the charging effect, but the droplets are easily adsorbed on the electrode by Coulomb forces. Therefore, it is necessary to find the optimal electrode-mounting distance. Patel et al. (2013, 2015) researched the effect of electrode shape and position on the charging effect. The results showed that the square electrode with an inner circular section has the best effect, and the suitable distance range from the electrode to the cross-section of the nozzle outlet is 2–3 mm. Maski and Durairaj (2010) studied the effect of charging voltage on droplet deposition and concluded that moderate application speed and high charging voltage contribute to droplet deposition. Yule et al. (1995) investigated the relationship between charging voltage and spray angle. The results showed that the spray angle was positively correlated with the charging voltage. Zhou et al. (2015) and Li et al. (2007) found that the charging voltage was the most important factor affecting the deposition of droplets on the abaxial surface of leaves. Lan et al. (2018) and Patel et al. (2013) studied the effect of different electrode materials on the charging ability. The results showed that the electrode with purple copper as the material had the best charging effect. So, with the application of induction charging method, suitable material and structure used in electrode design and its insulation, spray atomization methods, combined with external air assistance, the charging performance of electrostatic nozzles and the efficiency of pesticide deposition on leaves could be improved to reduce the environmental pollution by pesticide off-target loss (Shrimpton and Laoonual, 2006; Patel, 2016). Despite our abovementioned efforts to improve the performance of electrostatic spray, we get the fine charged droplets with adhesion ability that we need. What still needs our attention is that the electrostatic spray system may not be effective as it is affected by the complex operating environment of agriculture. Pascuzzi and Cerruto (2015) used the ESS electrostatic-induction nozzle in “tendone” vineyards. The activation of the electrostatic system produced a significant increase in the mean foliar deposit only on the lower layer, while it had no effect on the upper layer. Zhou et al. (2015) found that in the application of an air electrostatic nozzle, the adhesion ability of the charged droplets is weakened due to the charge decay, when the spraying distance is far. At the same time, the fine droplets are more prone to drift due to ambient winds when transported over long distances. Therefore, in addition to studying how to improve the performance of electrostatic spraying devices, we also need to study what working conditions to better utilize the effect of electrostatic spraying.

Additionally, some researchers (Shang et al., 2004; Wu et al., 2009; Zhou et al., 2019) conducted studies on operational parameters such as spray pressure, external airflow supply, application speed, and spray distance. Appah et al. (2019b) conducted studies that showed that the combined parameters of applied voltage, liquid flow pressure, and spraying height produced maximum charged spray swath and fine droplets. Patel et al. (2016a) found that external airflow had a significant effect on spray atomization and the distribution of external airflow field could affect the spray coverage. Maski and Durairaj (2010) concluded that both spray distance and application speed have an effect on droplet deposition efficiency. It was concluded that the optimization of the operating parameters can effectively improve the spray performance and deposition effect of the electrostatic nozzle (Patel et al., 2017).

From the above literature studies, it is clear that air-assisted electrostatic spraying can effectively improve the deposition of pesticide droplets in the crop canopy helping to improve the utilization rate of pesticides and pest control effects. However, further optimization and design are needed to the internal flow channel structure and electrode parameters of the nozzle to meet the actual requirements of pesticide application. Furthermore, the analysis of charge droplet deposition mechanism and its influencing factors is a hot research topic at present, but the main research focused on the influence of charge parameters on deposition with CMR as an indicator. The canopy characteristic parameters and spraying operation parameters have not been sufficiently studied, and the interaction effects between each parameter are still unclear. To solve the above situation, an improved air-assisted electrostatic nozzle with induction charging will be designed and optimized in this article. Spraying performance experiments will be conducted to obtain the appropriate operating parameters with good atomization and chargeability. The multi-factor orthogonal experiment will be designed and analyzed to ensure the influence of each factor on the droplet penetration and deposition, which could provide a technical reference for the optimization of operating parameters of electrostatic spraying. With these improvements and research, farmers could be guided to spray pesticides better, reduce pesticide usage, and increase pesticide utilization efficiency.

## 2 Materials and methods

### 2.1 Air-assisted electrostatic spraying system

**Figure 1:**
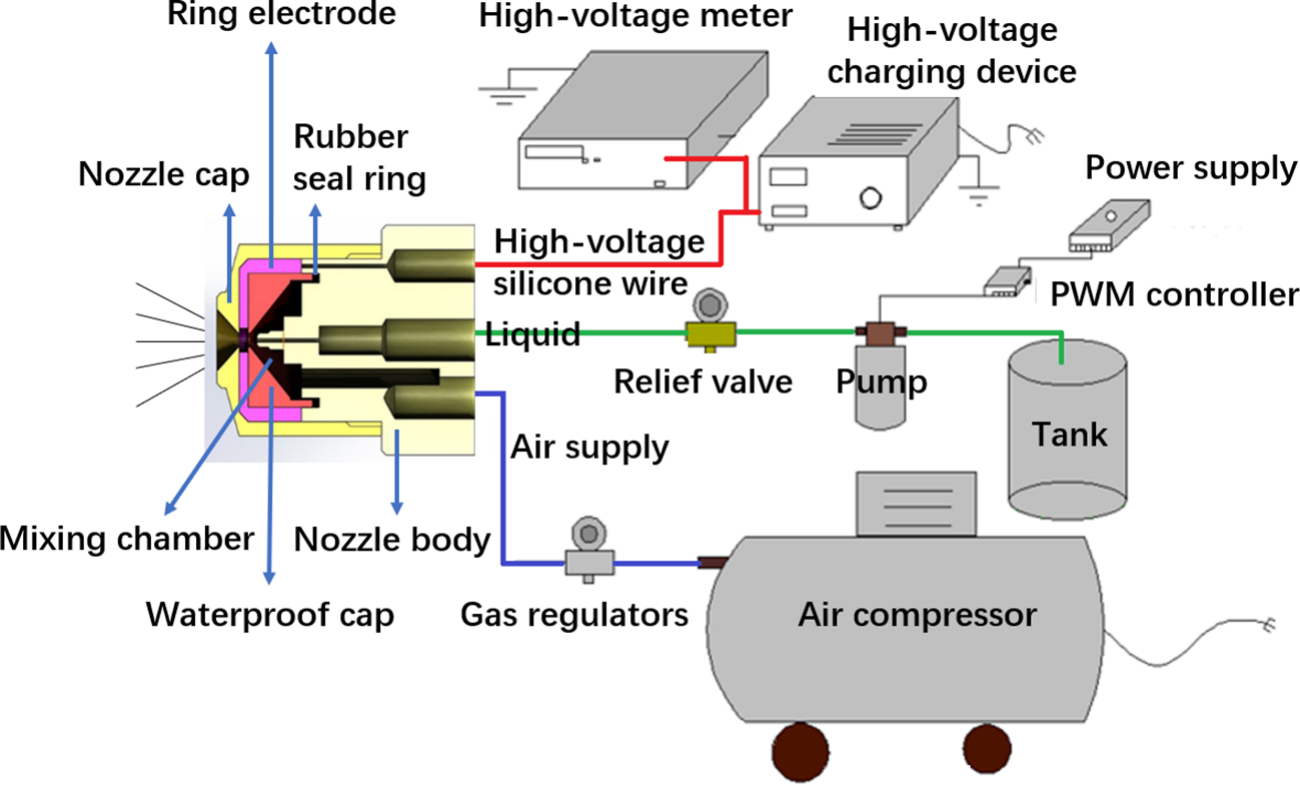
Schematic diagram of an air-assisted electrostatic spraying system.

An air-assisted electrostatic spraying system has been designed and developed as shown in the schematic diagram of **Figure 1**. It mainly consists of air-assisted induction electrostatic nozzle, high-voltage charging device, flow rate adjustment device, liquid supply device, and air supply device. The high-voltage charging device (GF-2A, Wuxi, China) is required to provide high voltage (Ultra Volt +20 kV, 1 mA, 20 W, for laboratory experiments only) for the charging of the conductive liquid, which was connected to the ring electrode inside the nozzle by an insulated wire. The charging of conductive liquid is based on the induction principle, which is the most reliable, safe, and experimentally proven method for efficient charging (Patel et al., 2017). A pulse width modulation (PWM) (Mingwei, Shenzhen, China) controller was used to control the flow rate of liquid from a tank. Liquid flows from a pump (Xishan DP-150, Shanghai, China) into a nozzle and is atomized by high-speed airflow. An air compressor (Model JB-750×3, Zhejiang, China) and a pressure regulator were used to obtain the required high-speed steady airflow. A high-voltage meter (Model 900B, Chroma Electronics (Shenzhen) Co. Ltd, China) is responsible for real-time monitoring and showing voltage variations. Both the high-voltage electrostatic device and the high-volt meter need to be well grounded.

**Figure 2:**
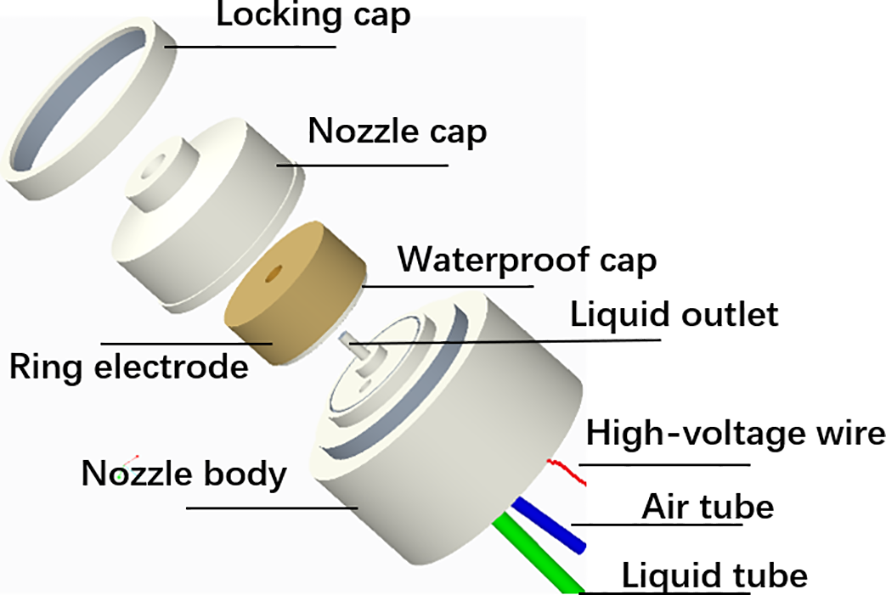
Structural diagram of an air-assisted electrostatic nozzle.

The electrostatic nozzle is a key component to achieve the atomization of pesticide liquid and charge of droplets in an electrostatic spraying system. The structure of the air-assisted electrostatic nozzle is shown in **Figure 2**. The internal structure and charging electrode parameters need to be considered in the design of the electrostatic nozzle, since they are critical to atomization performance and charge performance of the nozzle. The traditional hydraulic electrostatic nozzle has a problem of short spray distance, leakage, and reverse ionization causing short droplet transport distance and poor charging and deposition effect. To improve the above problems, we designed and optimized the nozzle structure and electrode parameters using a coaxial gas–liquid twin fluid, internal mixing, air-assisted atomization method. The charging of conductive liquid based on the induction principle was used, which is the most reliable and field-proven method for imparting charge efficiently (Patel et al., 2017).

In addition, we have improved the air-assisted electrostatic nozzle in terms of runner structure, electrode parameters, insulation, and waterproof measures. First, the diameter of the liquid outlet is one of the factors that determine the droplet size and atomization effect; the smaller the diameter, the finer the droplet. Considering the limitations of machine processing, we set the diameter of liquid outlet to 1 mm. Concurrently, we increased the size of the internal gas–liquid mixing chamber and ensured sufficient airflow at the nozzle outlet, which would be more beneficial to liquid atomization. The throat size is set to 3 mm. With a tapered shrinkage structure, the airflow creates a choking effect at the throat, which leads to a sharp increase in air velocity. In this way, the charged droplets were able to be carried away by the airflow quickly and did not adhere to the inner circle surface of the electrode improving charge capacity (Patel et al., 2016b, Patel et al., 2017). Second, a ring copper electrode with an inner circular section was used, and the distance from the nozzle exit to the electrode is 3 mm, close to the atomization zone, which were proven by Patel to be the suitable electrode parameters for charging (Patel et al., 2013, Patel et al., 2015). To avoid liquid droplets hitting the inner circle cross of the copper electrode, the diameter of the inner circular section is set at 5 mm. Third, we used an insulated waterproof cap and a rubber seal ring to form a grooved sealing structure to isolate the ring electrode from the gas–liquid mixing chamber avoiding leakage and reverse ionization caused by direct contact of the electrode with the conductive liquid. To improve the insulation and prevent electrical breakdown, the waterproof cap and nozzle cap are made of Teflon material.

### 2.2 Spraying performance experiment

The spraying performance of the electrostatic nozzle is essential to the quality of spraying operations. Good atomization and chargeability are required to ensure that the pesticide liquid is fully atomized, the droplets are fine and uniform with sufficient charge to adhere to the surface of leaves. In this study, we measured the CMR, droplet size, and distribution to evaluate the charge ability and atomization performance of the electrostatic nozzle. All the experiments were conducted at the Key Laboratory of Plant Protection Engineering in Jiangsu University, a closed laboratory with static air inside to prevent droplet spraying out of the test areas, with humidity of 68% and temperature of 25°C to provide ideal droplet evaporation simulating field conditions.

#### 2.2.1 Charging performance

In electrostatic spraying, the foremost aim is to provide a significant charge to spray droplets to deliver them effectively and efficiently to the intended target. The more electrostatic charge carried by the droplets, the better will be the performance in terms of increased uniformity, deposition efficiency, wraparound effect, and reduced off-target losses (Patel et al., 2022). The chargeability of the droplets, i.e., their capability to acquire charge, is evaluated based on the amount of electrostatic charge per unit mass of the droplet, called the CMR (Maski and Durairaj, 2010; Pascuzzi and Cerruto, 2015). The CMR is the most important parameter that defines the charge performance of the electrostatic nozzle. According to the above definition, the CMR could be calculated from the following relation [Equation 1]:


(1) $$CMR=\frac{q}{m}=\frac{it}{m}=\frac{i}{Q_{m}}$$

where i is the measured spray current (A), *Q_m_* is the mass flow rate of liquid (kg s^−1^), m is the mass of liquid collected in the beaker at a specific time, and q is the droplet charge (C).

**Figure 3:**
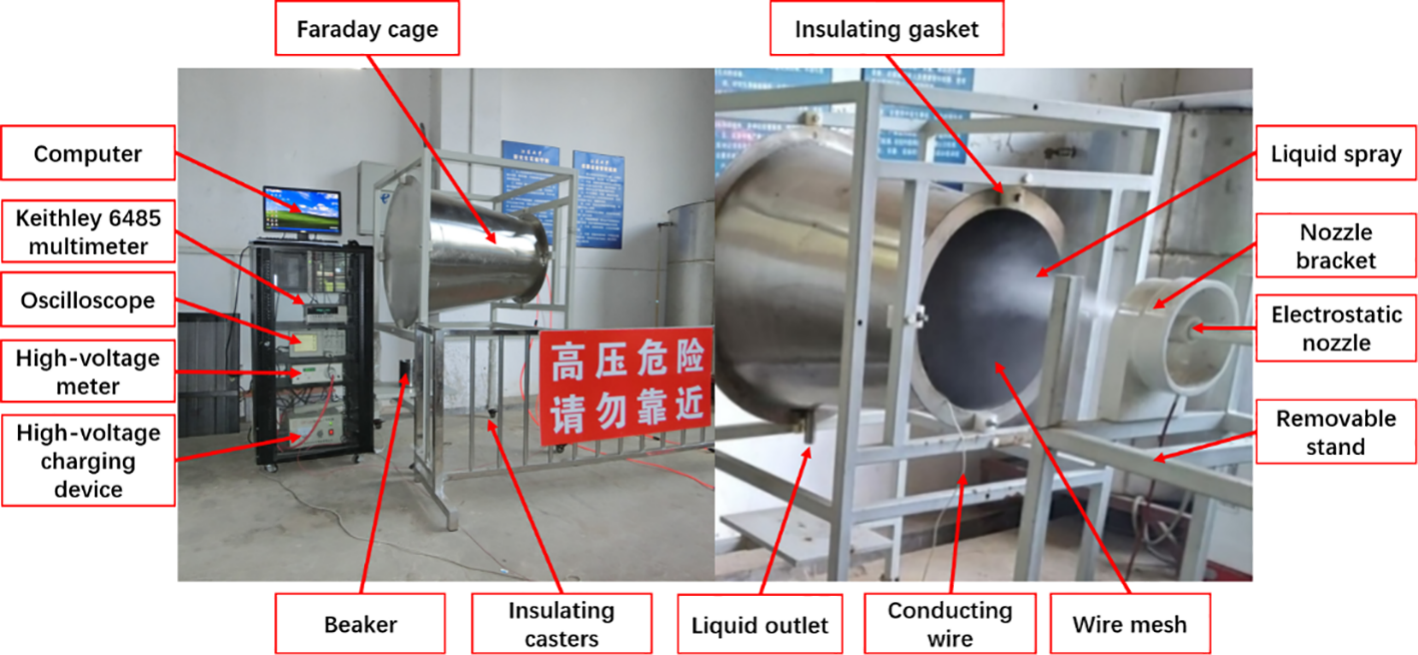
Charge-to-mass ratio measurement system.

The Faraday cage method was used in the CMR measurement; a specially designed Faraday cage was connected to the digital multimeter (Model No. 6485, Keithley A Tektronix Inc. Company, Ohio) via a conducting wire as show in **Figure 3**. The digital multimeter must be reliably grounded. The charge droplets contact the wire mesh of the Faraday cage and transfer the charge to the ground causing an electrical current, which was detected and measured by the digital multimeter in real-time. The current data was logged into ExceLINX (ELNX-852C04) software in a computer through a USB cable during the operation for data storage and analysis (Appah et al., 2019b). The charged liquid spray was collected at a specific time in a beaker and weighed by precision electronic balance.

The experiment was designed to be divided into two groups to measure the variation of CMR with charge voltage at different distances (0.1, 0.4, 0.7, and 1.0 m) and air pressure (0.5, 1.0, and 1.5 bar) conditions. The flow rate of the nozzle was 200 ml/min, and continuous spray and current measurement for 1 min (60 s). The charged voltage ranges from 0 to 10 kV, adjustable every 1 kV.

#### 2.2.2 Droplet size and distribution

Droplet size is one of the main factors affecting the deposition and distribution uniformity of pesticides on the target. Droplet size and distribution uniformity are identified as essential factors to evaluate the spray performance and deposition efficiency in agricultural spray application (Liao et al., 2020). Only with a suitable droplet size can more droplets be captured on the target surface and the better control effect of pests and diseases.

The volume median diameter (VMD) and the relative span (RS) were used to characterize the mean droplet size and the uniformity of droplet distribution in this research. The droplet size parameters (such as D_V0.1_, D_V0.5_, and D_V0.9_) were able to be measured and recorded by the Laser Particle Size Analyzer (LPSA) (model Winner 318, Shandong, China). The D_V0.5_ indicates that half of the volume of spray is in droplets smaller than this value (D_V0.5_ = VMD). With D_V0.1_, D_V0.5_, and D_V0.9_, the RS of the droplet spectrum can be calculated from Equation 2. RS was used to characterize the uniformity of droplet distribution; the smaller the RS values, the less variation there is between the size of the droplets in spray spectrum, and the more uniform the droplet distribution is (Ru et al., 2020).


(2) $$RS=\frac{\left[D_{V0.9}-D_{V0.1}\right]}{D_{V0.5}}$$

**Figure 4:**
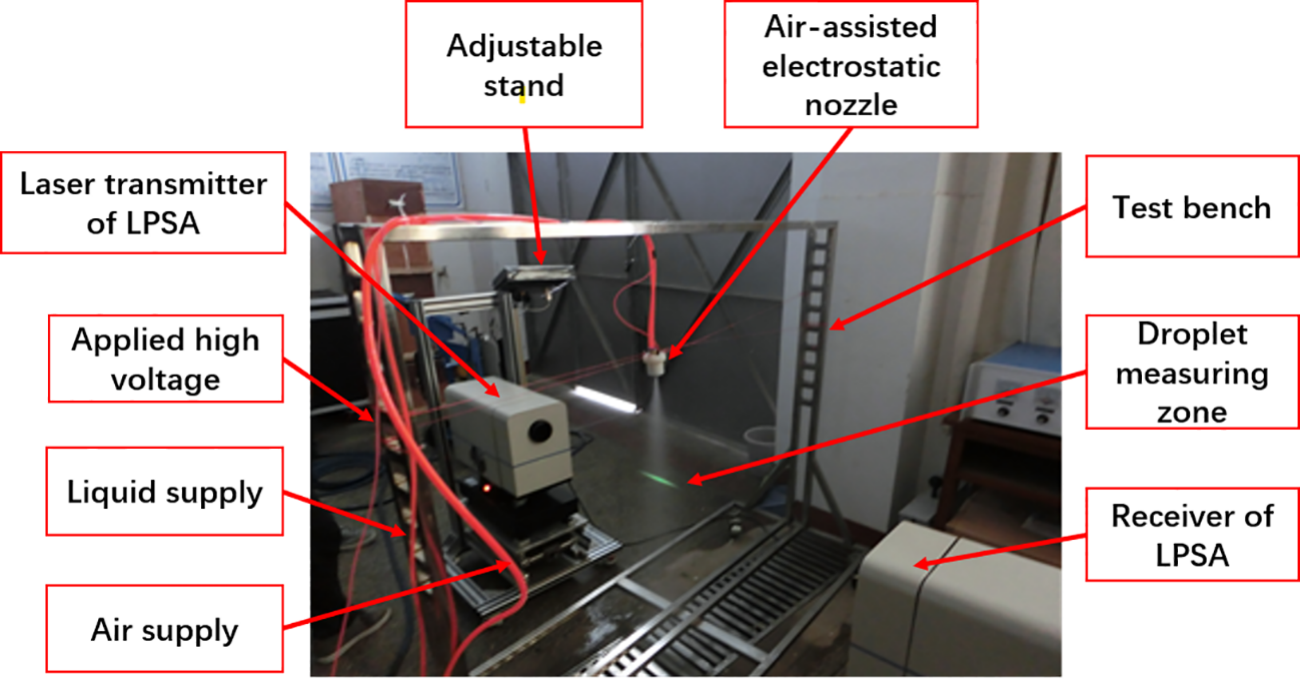
Experimental set-up to measure the droplet size.

The air-assisted electrostatic nozzle was fixed on the test bench, turning on the spray system, and adjusting the position of the LPSA so that the emitted laser passed through the center of the droplet cluster and reflected back to the receiver side. Tests need to be conducted in weak light environment to reduce the interference of ambient light. The height of the nozzle from the laser beam is kept at 0.5 m. The air pressure was set at 0.5 bar, 1.0 bar, and 1.5 bar, and the charged voltage was adjusted from 0 V to 10 kV. The experimental set-up is shown in **Figure 4**.

### 2.3 Experiment to study the deposition characteristic of charged droplet

**Figure 5:**
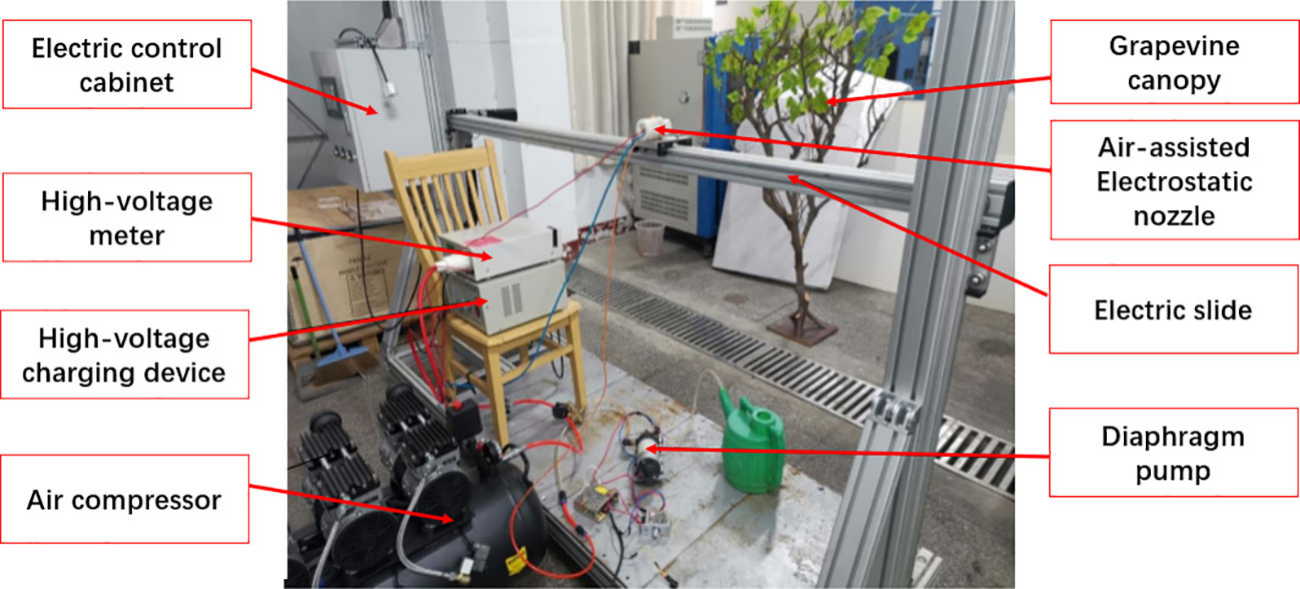
Experimental set-up to study spray deposition characteristics and canopy penetration.

The experiment was carried out inside the Key Laboratory of Plant Protection Engineering in Jiangsu University with a temperature of 25.1°C and relative humidity of 42%. In this experiment, the improved air-assisted electrostatic nozzle was used to conduct experimental research on spray penetration and deposition characteristics with the canopy of grapevine. The nozzle was fixed to the slide and moves with the slide at a speed of 1 m/s when spraying. Simulated leaves similar to the size of grape leaves were selected for canopy construction according to the characteristics of the grapevine. The leaf area index (LAI) were measured to characterize the canopy, which is referred to as the total one-sided area of leaf tissue per unit ground surface area. The area for each leaf was evaluated in the laboratory using a scanner (Lenovo M7605D) and a measuring software (Image Pro Plus, Media Cybernetics). The canopy size of the grapevine is 0.6 m (thickness) × 0.8 m (width) × 0.8 m (height). The leaf area index was set at three levels of 1, 1.5, and 2 by changing the arrangement and number of leaves. Three sampling points were selected inside the canopy at a distance of 0.3 m from the front of the canopy. Water-sensitive papers (WSPs) were arranged at each sample point to collect droplets during spraying. The WSPs were fixed by a double-ended clip with one end clamped to the branch of the grape canopy and the other end clamped to WSPs ensuring a fixed position of WSPs for each group of experiments. The experiment set-up is shown in **Figure 5**.

The WSPs needed to dry before collecting into labeled ziplock bags after each spraying test and were then numbered and scanned. It is essential to wear rubber gloves during the WSP collection process. A high definition scanner was used to obtain the 600-dpi image of scanned WSPs. The “DepositScan” software, which was developed by the USDA-ARS Application Technology Research Center, was applied to evaluate droplet size, droplet distribution, total droplet number, droplet density, amount of spray deposits, and percentage of spray coverage (Zhu et al., 2011). The scanned images were imported into the DepositScan for analysis, and the amount of droplet deposition per unit area of each WSP was derived. In the penetration experiment, the penetration was reflected by the average of the adaxial deposition captured by the WSP at three sampling points; in the abaxial deposition experiment, the WSP was arranged in the reverse direction at the original three sampling points. The abaxial droplet deposition was reflected by the average value of the abaxial droplet deposition.

The Equation 3 was used to convert the spot area to the actual droplet diameter (d, μm) in the DepositScan software is as follows (Zhu et al., 2011):


(3) $$d=0.95d_{s}^{0.910}$$

where, ***d_s_*** can be calculated from the following Equation 4


(4) $$d_{s}=\sqrt{\frac{4A}{\pi }}$$

and A is the spot area (μm^2^) acquired from ImageJ. The spot area was calculated from the number of spot image pixels divided by the scanning resolution. In this program, the scanning resolution was chosen up to 2,400 dots per inch (dpi), or 10.58 μm per pixel length, which would allow detection of a droplet that has a minimum diameter of 17 μm (Zhu et al., 2011; Ahmad et al., 2020). However, the image software cannot consider the impact of spread factor (Hoffmann and Hewitt, 2005). The final actual droplet diameter D can be calculated from the Equation 5 (Zhu et al., 2011),


(5) $$D=1.06A^{0.455}$$

To clarify the main factors influencing the experiment results and obtain the optimal combination of different factors at different levels with fewer experiments, a four-factor, three-level orthogonal experiment was designed. Considering the air pressure, charge voltage, spray distance, and leaf area index as the factors of the orthogonal experiment, orthogonal experiment factor levels are shown in **Table 1**. Analysis of variance (ANOVA), performed using the SPSS Statistical Software Package (version 22.0, IBM, New York, USA), was used to determine the effect of each single factor and the interaction term on penetration and adhesion, and select the optimal operating parameters accordingly. The least significant difference (LSD0.05) test was applied to separate treatment means after F-test indicated the statistical significance at a probability level of 0.05.

**Table 1:** Orthogonal experiment factor levels.

Number	A (air pressure/bar)	B (spray distance/m)	C (leaf area index)	D (charge voltage/V)
1	0.5	0.2	1.0	0
2	1.0	0.6	1.5	2,500
3	1.5	1.0	2.0	5,000

Through the pre-test, it was found that in the droplet penetration experiment, there was a significant difference in droplet deposition volume of leaf adaxial when the leaf area index was increased from 1 to 2 at different spraying distances; similarly, there was a significant difference also when the air pressure was increased from 0.5 to 1.5 bar. Thus, the interaction term air pressure × distance and the interaction term distance × leaf area index were both included in the experimental design. The droplet penetration experiment was performed using an $L_{27}(3^{13})$ orthogonal design. The orthogonal test head is shown in **Table 2**.

**Table 2:** The orthogonal experiment head of penetration experiment.

**Number**	1	2	3	4	5	6	7	8	9	10	11	12	13
**Factor**	A	B	(A × B)1	(A × B)2	C			(B × C)1		D	(B × C)2		

The results of the pre-test showed that the effect of charge voltage on droplet deposition of leaf abaxial was not absolute. There was a significant difference in the incremental amount of droplet deposition of leaf abaxial induced by an increase in charge voltage, with different air pressure and spray distance conditions, which proved that there were significant interactions between air pressure and charge voltage, and spray distance and charge voltage in the droplet deposition of the abaxial leaf experiment. Thus, both the interaction term air pressure × charge voltage and the interaction term spray distance × charge voltage were incorporated into the experimental design to investigate the factors affecting the abaxial leaf adhesion. The experiment was performed using an $L_{27}(3^{13})$ orthogonal design. The orthogonal test head is shown in **Table 3**.

**Table 3:** The orthogonal experiment head of deposition on abaxial leaf.

**Number**	1	2	3	4	5	6	7	8	9	10	11	12	13
**Factor**	B	D	(B × D)1	(B × D)2	A			(A × D)1		C	(A × D)2		

## 3 Results and discussion

### 3.1 Effect of air pressure, charge voltage, and spray distance on the CMR

**Figure 6:**
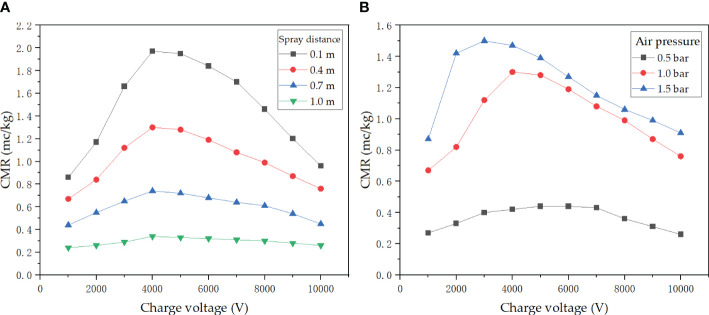
**(A)** Effect of spray distance and charge voltage on CMR. **(B)** Effect of air pressure and charge voltage on CMR.

**Figure 6A** reflects the results of CMR with variation of air pressure and spray distance, at an air pressure of 1.0 bar and a flow rate of 200 ml/min. The results show that the CMR increased by 127.8% from 0.86 to 1.97 mC/kg as the charge voltage increases from 1,000 to 4,000 V at a spray distance of 0.1 m. The CMR is on a downward tendency as the charge voltage increases from 5,000 to 10,000 V. The CMR ranges from 0.44 to 0.74 mC/kg at a spray distance of 1 m. When the spray distance is 1 m, the maximum CMR is only 0.34 mC/kg. It is clear that the CMR has a significant attenuation with the increase in spray distance.

**Figure 6B** describes the variation of CMR with charge voltage under different air pressure conditions at a spray distance of 0.4 m and a flow rate of 200 ml/min. The result shows that the CMR reaches a maximum of 1.5 mC/kg at a charge voltage of 3,000 V, when the air pressure is 1.5 bar. The CMR reaches its maximum at a voltage of 4,000 V, but decreases as the charge voltage increases after exceeding 4,000 V. When the air pressure is at 0.5 bar, the CMR is small, ranging from 0.2 to 0.4 mC/kg.

With the analysis of the above results, it is clear that the CMR has a critical peak when the charge voltage is between 3,000 and 4,000 V. The CMR conversely decreases as the charge voltage continues to increase. Therefore, for the electrostatic nozzle, “the higher the charging voltage, the better the charging effect” does not apply. It is impractical to increase the CMR by increasing the charge voltage alone. It will increase the electrical power consumption and costs, which are not beneficial to agricultural pesticide application. Applied air pressure also has a significant effect on the CMR, especially when the CMR increases significantly when the air pressure increases and reaches a peak at a relatively small charge voltage. It is due to the increase in air pressure, which improves the atomization effect of the nozzle, which leads to the formation of more small droplets. The same amount of liquid with smaller-sized droplets will have more charge in comparison to bigger-sized droplets (Patel et al., 2016b, Patel et al., 2017). In addition, the appropriate spray distance needs to be selected because the charge would decay with time and distance in the transport of charged droplets in the air. Therefore, optimization of design and performance parameters is important for optimum performance.

### 3.2 Effect of air pressure and charge voltage on droplet size and distribution

**Figure 7:**
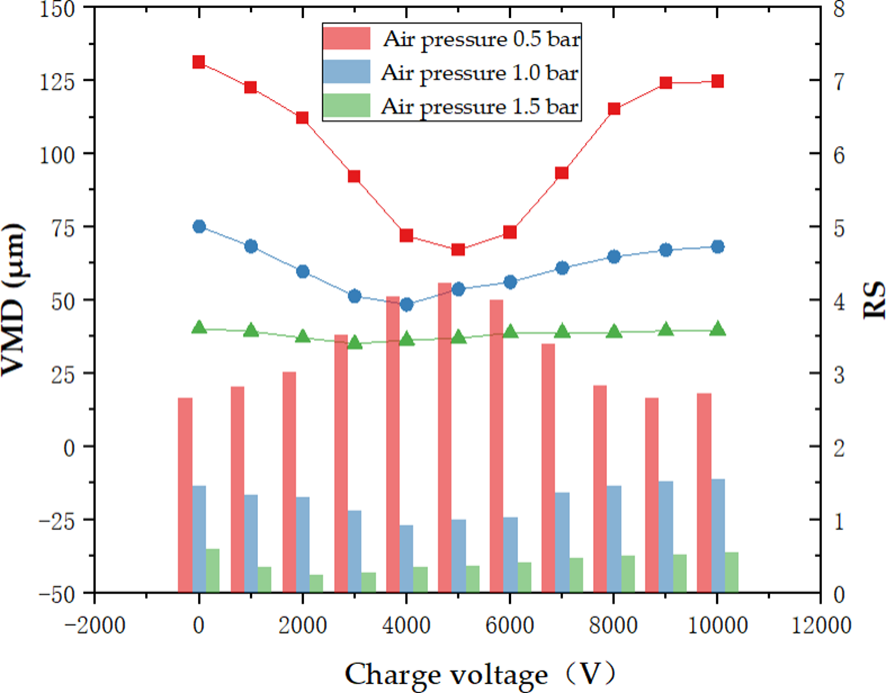
Effect of air pressure and charge voltage on VMD and RS.

**Figure 7** shows the results of VMD and RS of the droplets with variation in the charge voltage and air pressure. In **Figure 7**, the VMD showed a significant change with the variation in charge voltage when the air pressure is 0.5 bar. The VMD achieves a minimum value of 67.025 μm at a voltage of 5,000 V, which is a 48.9% reduction from 131.153 μm without electrostatic charge. When the air pressure is 1.5 bar, the VMD ranges from 35.102 to 40.232 μm. The charge voltage has a relatively smaller effect on VMD at this time.

From the results, it can be seen that under the same charging conditions, the VMD decreases with the increase in air pressure. From the red line, the droplet size is most obviously affected by the charge voltage, and the maximum decrease is 48.9%, when the air pressure is 0.5 bar. It was concluded that the charge effect would affect the atomization effect of the droplets. The electrostatic charging could affect the secondary atomization of the liquid droplets, especially for large droplets over 100 μm, which creates more small droplets and leads to a significant reduction in VMD. However, when the air pressure increases, the atomization of liquid droplets is intensified by the impact force and shearing effect of the airflow. The liquid droplets are fully atomized and evenly broken into smaller droplets at this time. The VMD ranges smoothly from 25 to 50 μm. The atomization and break-up of these fine droplets by electrostatic forces become limited.

The relative span (RS) was used to characterize the uniformity of droplet distribution in this research. The smaller the RS, the less variation there is between the size of the droplets in spray spectrum. In **Figure 7**, the RS shows an increasing trend as the charge voltage increase at an air pressure of 0.5 bar. Liquid droplets break up into small droplets under electrostatic action, which reduces the VMD but increases the particle size spectrum. This results in an RS much higher than 1. When the air pressure is 1.0 bar, the RS tends to be close to 1 under the action of electrostatic forces. The closer the RS is to 1, the closer the droplet size distribution is to the normal distribution, that is, the more uniform the droplet distribution is (Ru et al., 2020). Additionally, in terms of the results of VMD and RS, electrostatic atomization works with particle sizes approximately 50–75 μm under the above experimental conditions. Air atomization or hydraulic atomization plays a leading role in some situations.

For most crops, the optimum particle size suitable for deposition ranges from 20 to 50 μm. In electrostatic spray, the fine droplets have better charge ability and were able to overcome the drift due to their light mass with the help of electrostatic force and air (Patel et al., 2017). The higher the air pressure, the smaller the VMD and RS, and the smaller the particle size, which indicate that the nozzle has good atomization effect and the droplet size distribution is uniform. However, pesticide droplets with too small particle size are volatile and more prone to drift from the actual target, which are not suitable for agricultural spraying operation requirements (Patel et al., 2022). Furthermore, the higher air pressure can also lead to increasing gas power consumption, which can increase agricultural costs and discourage the design and development of spraying equipment.

### 3.3 Experiment to study the deposition characteristic of charged droplet

For electrostatic spraying, interactions between the various factors affecting spray deposition may exist. Considering the interactions provides a more description of the relationship between spraying parameters and spraying effect, making it easier to obtain the optimal combination parameters for spraying operations.

#### 3.3.1 Effect of spraying parameters and canopy characteristics on droplet penetration

The results of the penetration orthogonal experiment are shown in **Table 4** based on the orthogonal experiment table.

**Table 4:** Results of orthogonal experiments for droplet penetration.

Numbers	Factors								Droplet deposition (μl/cm^2^)
	A	B	(A × B)1	(A × B)2	C	(B × C)1	D	(B × C)2	
1	1	1	1	1	1	1	1	1	0.998
2	1	1	1	1	2	2	2	2	0.710
3	1	1	1	1	3	3	3	3	0.480
4	1	2	2	2	1	2	2	3	0.691
5	1	2	2	2	2	3	3	1	0.422
6	1	2	2	2	3	1	1	2	0.288
7	1	3	3	3	1	3	3	2	0.346
8	1	3	3	3	2	1	1	3	0.154
9	1	3	3	3	3	2	2	1	0.115
10	2	1	1	3	1	1	2	1	1.267
11	2	1	2	3	2	2	3	2	1.018
12	2	1	3	3	3	3	1	3	0.826
13	2	2	1	1	1	2	3	3	0.960
14	2	2	2	1	2	3	1	1	0.672
15	2	2	3	1	3	1	2	2	0.442
16	2	3	1	2	1	3	1	2	0.499
17	2	3	2	2	2	1	2	3	0.269
18	2	3	3	2	3	2	3	1	0.134
19	3	1	3	2	1	1	3	1	1.325
20	3	1	3	2	2	2	1	2	1.133
21	3	1	3	2	3	3	2	3	0.941
22	3	2	1	3	1	2	1	3	1.075
23	3	2	1	3	2	3	2	1	0.806
24	3	2	1	3	3	1	3	2	0.576
25	3	3	2	1	1	3	2	2	0.595
26	3	3	2	1	2	1	3	3	0.326
27	3	3	2	1	3	2	1	1	0.211
*T*_1_	4.204	8.698	7.371	5.394	7.756	5.645	5.856	5.950	
*T*_2_	6.087	5.932	4.492	5.702	5.510	6.047	5.836	5.607	
*T*_3_	6.988	2.649	5.416	6.183	4.013	5.587	5.587	5.722	

ANOVA was performed on the experiment results in **Table 4**. The results of the ANOVA are shown in **Table 5**.

**Table 5:** Analysis of variance of penetration experiment results.

Variance term	Sum of squared deviations	Degrees of freedom	F-value	*F*_0.95_ (*df_j_*, *df_e_*)
A	0.44847	2	186.94	4.103
B	2.03777	2	849.42	4.103
C	0.788745	2	328.78	4.103
D	0.005011	2	2.09	4.103
A×B	0.515344	4	107.41	3.478
B×C	0.02076	4	4.33	3.478

Referring to the table of F-distribution, F_0.05_ (2,10) = 4.10 and F_0.05_ (4,10) = 3.48; the value of *F_A_*, *F_B_*, *F_C_*, *F*_A*B_ exceed the threshold values, which shows that air pressure, spray distance, canopy leaf area index, and the interaction term air pressure × spray distance all had a significant effect on drop penetration, with the factor of spray distance having a determining effect on the results, followed by leaf area index, air pressure, and air pressure × spray distance. The results of the experiment are as follows: T_A3_ > T_A2_ > T_A1_, T_B3_< T_B2_< T_B1_, T_C3_< T_C2_< T_C1_. Overall, droplet penetration increased with increasing air pressure and decreased with increasing spray distance and leaf area index. Analyzing the above results, we found the following.

The effect of air pressure on droplet penetration is affected by changes in spray distance. When the distance is closer, the effect of air pressure on droplet penetration is significant; when the distance increases, the effect of air pressure on penetration is weakened. This is due to the wind speed generated at different air pressure decay at different rates with the increase in spray distance. This is shown by the fact that the higher the air pressure, the faster the wind speed decay.

The effect of charge voltage on droplet penetration was not significant. Voltage affects the charging effect of droplets. Charged droplets are more likely to be adsorbed on the leaves, which improves droplet attachment on the leaf surface. However, the effect on the deposition inside the canopy was not significant. It suggests that the “surround adsorption effect” of charged droplets under electric field force are more easily adsorbed on the leaf surface, but there are limitations with this effect. On the one hand, the charge of the charged droplets decays due to the effect of distance; on the other hand, due to the influence of the thin canopy, more charged droplets are adsorbed by the leaves on the outside of the canopy. Accordingly, the amount of droplets entering the inside canopy will decrease. Hence, air assistance is required to help disturb the surface canopy and increase the chances of charged droplets reaching the inner canopy.

#### 3.3.2 Effect of spraying parameters and canopy characteristics on droplet deposition of abaxial leaf

The results of abaxial droplet deposition are shown in **Table 6**.

**Table 6:** Results of orthogonal experiments for droplet deposition on abaxial leaf.

Numbers	Factors								Droplet deposition (μl/cm^2^)
	B	D	(B × D)1	(B × D)2	A	(A × D)1	C	(A × D)2	
1	1	1	1	1	1	1	1	1	0.015
2	1	1	1	1	2	2	2	2	0.018
3	1	1	1	1	3	3	3	3	0.019
4	1	2	2	2	1	2	2	3	0.012
5	1	2	2	2	2	3	3	1	0.017
6	1	2	2	2	3	1	1	2	0.045
7	1	3	3	3	1	3	3	2	0.010
8	1	3	3	3	2	1	1	3	0.028
9	1	3	3	3	3	2	2	1	0.032
10	2	1	1	3	1	1	2	1	0.011
11	2	1	2	3	2	2	3	2	0.014
12	2	1	3	3	3	3	1	3	0.016
13	2	2	1	1	1	2	3	3	0.008
14	2	2	2	1	2	3	1	1	0.015
15	2	2	3	1	3	1	2	2	0.037
16	2	3	1	2	1	3	1	2	0.008
17	2	3	2	2	2	1	2	3	0.020
18	2	3	3	2	3	2	3	1	0.027
19	3	1	3	2	1	1	3	1	0.006
20	3	1	3	2	2	2	1	2	0.013
21	3	1	3	2	3	3	2	3	0.013
22	3	2	1	3	1	2	1	3	0.007
23	3	2	1	3	2	3	2	1	0.009
24	3	2	1	3	3	1	3	2	0.020
25	3	3	2	1	1	3	2	2	0.005
26	3	3	2	1	2	1	3	3	0.012
27	3	3	2	1	3	2	1	1	0.014
*T*_1_	0.196	0.125	0.115	0.143	0.082	0.194	0.161	0.146	
*T*_2_	0.156	0.170	0.154	0.161	0.146	0.145	0.157	0.170	
*T*_3_	0.099	0.156	0.182	0.147	0.223	0.112	0.133	0.135	

ANOVA was performed on the experiment results in **Table 6**. The results of the ANOVA are shown in **Table 7**.

**Table 7:** Analysis of variance of droplet deposition experiment on abaxial leaf.

Variance term	Sum of squared deviations	Degrees of freedom	F-value	*F*_0.95_ (*df_j_*, *df_e_*)
A	0.0011080	2	40.03	4.103
B	0.0005280	2	19.07	4.103
C	0.0000509	2	1.84	4.103
D	0.0001180	2	4.26	4.103
A×D	0.0004492	4	8.11	3.478
B×D	0.0002718	4	4.91	3.478

From the above results, *F_A_*, *F_B_*, *F_D_*, *F*_A*D_, *F*_B*D_ exceed the critical value. This suggests that air pressure, spray distance, charge voltage, the interaction terms air pressure × charge voltage and spray distance × charge voltage all have a significant effect on droplet deposition of leaf abaxial. The degree of influence of each factor on the adhesion effect on the abaxial leaf is in the order of air pressure, spray distance, air pressure × charge voltage, spray distance × charge voltage, and charge voltage. During the spraying, the droplets move with the direction of the spray and are more likely to attach to the surface of the adaxial leaf with relatively little deposition on the abaxial surface of the leaf. The attachment of the charge droplets on the abaxial leaf is mainly due to the perturbation of the airflow and the electric field force on the charged droplets together. The interaction between factors was more significant in the droplet deposition experiment on the abaxial leaf.

According to the above results: T_A3_ > T_A2_ > T_A1_, T_B3_< T_B2_< T_B1_, T_C3_< T_C2_< T_C1,_ T_D2_ > T_D3_ > T_D1_. It can be seen that droplet deposition of the abaxial leaf increased with increasing air pressure and decreased with increasing spray distance and leaf area index. When the charge voltage is taken as 2,500 V, there is maximum droplet deposition on the abaxial leaf, which is higher than that of the case without charging. When the air pressure increases from 0.5 to 1.5 bar, we got a maximum deposition of 0.045 μl/cm^2^. This is due to the increased air pressure improving droplet delivery efficiency and penetration, while increased disturbance of leaves makes it easier for droplets to reach the inside canopy. Furthermore, the increase in air pressure improves the atomization and charging effect of liquid droplets. The CMR of the charge droplets is higher and easier to be adsorbed on the abaxial leaf by electric field forces under the condition of constant charge voltage. Additionally, the velocity of the charged droplets decreased when they reach the canopy as the spray distance increased from 0.2 to 1 m, which is more difficult for the droplets to reach the inside canopy. At the same time, the charge of the droplets decreases as the distance increases, and the CMR of the droplet decreases after reaching the inside canopy as the spraying distance increases leading to a decrease in droplet deposition on the abaxial leaves.

We suggest that there is an interaction among the factors of air pressure, charge voltage, and spray distance. For example, comparing the results of experiment groups 15 and 16, at the same spray distance of 0.6 m, the deposition at a voltage of 2,500 was 0.037 μl/cm^2^, which is much greater than the deposition at a voltage of 5,000 V of 0.008 μl/cm^2^. On the one hand, factors affecting the charging effect include the morphology of charged droplets at the nozzle exit, in addition to the charge voltage, electrode shape, and size (Patel et al., 2015). Specifically, when the air pressure is low, the atomization effect is not good, the droplet size is large, and the charging effect is not ideal; when the air pressure is increased and the droplet size becomes smaller, the charging effect of the droplets becomes better. Therefore, there is an interaction between air pressure and charged voltage on the charging effect, and this interaction is also reflected in the adhesion effect on the abaxial leaf. On the other hand, the effect of charged voltage on the charging effect is not linear; with the increase in voltage, the CMR increases before it reaches a peak and then gradually decreases, which is due to the arc discharge phenomenon generated by a high voltage (Zhou et al., 2018). Moreover, there is also an interaction between charge voltage and spray distance due to the different charging effects. When the spray distance is close, there is less charge decay of the charged droplets. Therefore, when the spraying distance is close, the charge voltage has a significant effect on the deposition of the abaxial leaf, and when the spraying distance is far, the adjustment of the charge voltage does not produce a significant change in the deposition effect.

In summary, to improve the deposition efficiency, the CMR should not only be increased by increasing the voltage but also the interactive effects of other spraying parameters and canopy characteristics on deposition should be considered. This study can provide recommendations for the specific application and implementation of electrostatic spraying in agricultural application. According to the results and analysis, the significance of the effect of each factor on droplet penetration, in descending order, is as follows: spray distance, leaf area index, air pressure, and air pressure × spray distance. Since charge voltage has no significant effect on droplet penetration, temporarily setting the charge voltage as 0 V, the optimal solution is obtained as *A*_3_ *B*_1_ *D*_1_ (air pressure 1.5 bar, spray distance 0.2 m, charge voltage 0 V). The significance of the effect of each factor on abaxial leaf deposition, in descending order, is as follows: air pressure, spray distance, air pressure × charge voltage, spray distance × charge voltage, and charge voltage. Obviously, air pressure and spray distance are still the priority. In view of the fact that there is some significant effect of charge voltage on the deposition of abaxial leaves, however, the deposition effect with a 2,500-V charge voltage is better than that of 5,000 V at an air pressure of 1.5 bar. By combining the above analysis and results, the final optimal solution is obtained as *A*_3_ *B*_1_ *D*_2_ (air pressure 1.5 bar, spray distance 0.2 m, charge voltage 2,500 V).

This improved air-assisted electrostatic nozzle in this study does not have a particularly higher CMR compared to the advanced air-induced air-assisted electrostatic nozzle designed by Patel, whose droplets are electrified to more than 10 mC/kg CMR by a charging voltage less than 2.5 kV at a liquid flow of 150 ml/min (Patel et al., 2017). However, in actual orchard spraying operations, we need to avoid excessive consumption of compressed gas during multi-nozzle orchard spraying as well as the requirements of high air pressure on the supporting gas supply equipment. Furthermore, our improved air-assisted electrostatic nozzle only requires a maximum air pressure of 1.5 bar, which is only under half of the air pressure compared to the electrostatic nozzle of Patel. With the same 2,500-V charge voltage, our improved air-assisted electrostatic nozzle can have 1.5 mC/kg of CMR at an air pressure of 1.5 bar, one adaxial droplet deposition of 0.941 μl/cm^2^, and another abaxial droplet deposition of 0.045 μl/cm^2^ under the above conduction, which are not less than the droplet deposition using the nozzle of Patel (with maximum adaxial droplet deposition of 0.83 μl/cm^2^ and maximum abaxial droplet deposition of 0.125 μl/cm^2^). We will conduct further field experiments to validate our equipment and improve our nozzle.

## 4 Conclusions

In this article, an improved air-assisted electrostatic nozzle with induction charging was designed, and an air-assisted electrostatic spraying system was built to conduct the spraying performance experiment of the nozzle. The objective of this study is to obtain the appropriate operating parameters of the electrostatic spraying system combination that maximizes chargeability and uniform droplet distribution and enhances droplet deposition in electrostatic pesticide application through exploring the general regulations of the effect of different operating parameters on the spraying performance. Furthermore, we designed a multi-factor orthogonal experiment and analyzed the impact mechanisms on droplet deposition and penetration characteristics under different operating parameters and canopy characteristics conditions.

The results of the spraying performance experiment proved that the improved air-assisted electrostatic nozzle has good atomization and charging effects. The spraying system has good atomization effect of droplets under the condition of air pressure of 0.5–1.5 bar. An excellent CMR (1.97 mC/kg) is achieved for efficient working of the electrostatic spraying processes with a charging voltage of 4,000 V. The CMR increased by 127.8% from 0.86 to 1.97 mC/kg as the charge voltage increases from 1,000 to 4,000 V at an air pressure of 1.0 bar and a flow rate of 200 ml/min. For the electrostatic nozzle, “the higher the charging voltage, the better the charging effect” does not apply. It is impractical to increase the CMR by increasing the charge voltage alone. It will increase the electrical power consumption and costs, which are not beneficial to agricultural pesticide application.

The electrostatic charging could affect the secondary atomization of the liquid droplets, especially for large droplets over 100 μm, which creates more fine droplets and leads to a significant reduction in VMD. When the air pressure is 1.0 bar, RS tends to be close to 1 under the action of electrostatic forces. Furthermore, the excessive air pressure can also lead to increasing gas power consumption, which can increase agricultural costs and discourage the design and development of spraying equipment. Considering all the above situation, for optimal droplet penetration and abaxial leaf deposition, option *A*_3_ *B*_1_ *D*_2_ is recommended.

These studies can provide technical references for the optimization of operating parameters of electrostatic spraying systems. The appropriate operation parameters could improve the pesticide droplet deposition and the penetration, thereby achieving a good control effect of pests and diseases. It can also guide the farmers to spray pesticides better reducing the pesticide usage and improving the pesticide utilization efficiency.

## Data availability statement

The original contributions presented in the study are included in the article/supplementary material. Further inquiries can be directed to the corresponding author.

## Author contributions

HZ: Conceptualization, Data curation, Formal analysis, Writing – original draft. MO: Conceptualization, Writing – review & editing. XD: Writing – review & editing. WZ: Data curation, Formal analysis, Writing – original draft. SD: Writing – original draft. WJ: Conceptualization, Writing – review & editing.
